# Cloning of a novel inhibin alpha cDNA from rhesus monkey testis

**DOI:** 10.1186/1477-7827-2-71

**Published:** 2004-10-07

**Authors:** Daniel J Bernard, Teresa K Woodruff, Tony M Plant

**Affiliations:** 1 Department of Neurobiology and Physiology, Northwestern University, 2205 Tech Drive, Evanston, IL 60208, USA; 2 Center for Biomedical Research, Population Council and The Rockefeller University, 1230 York Ave., New York, NY 10021, USA; 3 University of Pittsburgh School of Medicine, Departments of Cell Biology and Physiology, and Obstetrics, Gynecology and Reproductive Sciences, 3550 Terrace Street, Pittsburgh, PA 15261, USA

## Abstract

**Background:**

Inhibins are dimeric gonadal protein hormones that negatively regulate pituitary FSH synthesis and secretion. Inhibin B is produced by testicular Sertoli cells and is the primary circulating form of inhibin in most adult male mammals. Inhibin B is comprised of the inhibin alpha subunit disulfide-linked to the inhibin/activin betaB subunit. Here we describe the cloning of the cDNAs encoding these subunits from adult rhesus monkey testis RNA.

**Methods:**

The subunit cDNAs were cloned by a combination of reverse transcriptase polymerase chain reaction (RT-PCR) and 5' rapid amplification of cDNA ends (RACE) RT-PCR from adult rhesus monkey testis RNA.

**Results:**

Both the inhibin alpha and betaB subunit nucleotide and predicted protein sequences are highly conserved with other mammalian species, particularly with humans. During the course of these investigations, a novel inhibin alpha mRNA isoform was also identified. This form, referred to as rhesus monkey inhibin alpha-variant 2, appears to derive from both alternative transcription initiation as well as alternative splicing. rmInhibin alpha-variant 2 is comprised of a novel 5' exon (exon 0), which is spliced in-frame with exon 2 of the conventional inhibin alpha isoforms (variant 1). Exon 1 is skipped in its entirety such that the pro-alpha and part of the alpha N regions are not included in the predicted protein. rmInhibin alpha -variant 2 is of relatively low abundance and its biological function has not yet been ascertained.

**Conclusion:**

The data show that the predicted inhibin B protein is very similar between monkeys and humans. Therefore, studies in monkeys using recombinant human inhibins are likely to reflect actions of the homologous ligands. In addition, we have observed the first inhibin alpha subunit mRNA variant. It is possible that variants will be observed in other species as well and this may lead to novel insights into inhibin action.

## Background

The inhibins are dimeric gonadal protein hormones that negatively regulate pituitary FSH synthesis and secretion [1,2]. Inhibins are comprised of an α subunit (inhibin α) and one of two inhibin β subunits (inhibin βA or inhibin βB). In adult male mammals, inhibin B (α-βB dimer) appears to be the primary circulating form of the hormone, whereas females produce both inhibin A and B and do so in discordant fashion across the reproductive cycle [3-15]. One exception to this general pattern is in rams, where inhibin A appears to be the primary circulating form [16]. Historically, investigations of inhibin action have relied principally upon recombinant preparations of inhibin A because inhibin B has not been available in sufficient quantities to permit *in vivo *studies of its role in the negative feedback regulation of gonadotropin secretion [17,18]. Because inhibin B is the biologically relevant ligand in male primates, this has placed some constraints on our understanding of inhibin action in these animals. For this reason, we cloned the inhibin B subunit cDNAs from adult monkey testis as a requisite first step to producing recombinant monkey inhibin B. In the course of cloning the monkey inhibin α subunit, we identified a novel transcript, which has not been observed in other species. In this paper, we describe the new transcript called rhesus monkey inhibin α-variant 2.

## Methods

### RNA extraction

Total RNA was extracted from frozen testis samples of two adult male rhesus monkeys (*Macaca mulatta*) (#1861 and #2333) using Trizol following the manufacturer's instructions (Invitrogen, Carlsbad, CA). RNA was dissolved in diethyl pyrocarbonate-treated H_2_O and quantified by spectrophotometry. Animals were treated in accordance with institutional and federal guidelines.

### Reverse transcriptase polymerase chain reaction (RT-PCR)

**Figure 3:**
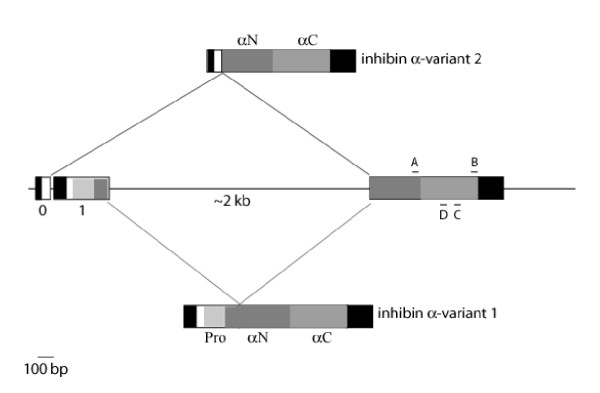
**Rhesus monkey inhibin α gene structure. **Schematic representation of the genomic organization of the inhibin α subunit in rhesus monkey. Boxed regions reflect exons and the intervening straight line is the intron (the 2 kb is an estimate based on the 2051 bp intron in humans). Black boxes reflect 5' and 3' UTRs. White boxes reflect sequences encoding the signal peptide (in inhibin α-variant 1) or a domain of unknown function (inhibin α-variant 2). The other shaded regions correspond to the pro-α, αN, and αC domains of the inhibin α prepro-hormone and are labeled in the figure. Inhibin-α variant 1 is the canonical inhibin α mRNA described previously in other species and is produced through splicing of exons 1 and 2. Inhibin α-variant 2 is produced through splicing of the novel exon 0 and exon 2. In this latter form, the entirety of exon 1 is removed, in addition to intron 1. An A to G transition in the monkey genomic sequence (relative to the human sequence) at the end of exon 0 appears to introduce a GT 5' splice donor site, which is absent in other species. This allows the splicing event observed in inhibin α-variant 2 when an upstream transcription start site is utilized. This variant is not predicted to exist in other species and also appears to be rare in monkey. The approximate positions of the primers used in different RT-PCR analyses are shown. A and B refer to the forward and reverse primers, respectively, designed to amplify the final 465 bp of the open reading frame. C and D are the outer and inner gene specific reverse primers used in the 5' RACE procedure.

Contaminating genomic DNA was removed from RNA samples using RQ1 DNase (Promega) following standard protocols. Four μg of DNased RNA (from #1861) was reverse transcribed into cDNA using 100 ng random hexamer primers and 100 U MMLV-RT (Promega). Four hundred ng of cDNA was subjected to PCR to amplify part of the αN domain and the entirety of the mature domain (αC) of the inhibin α subunit using the following primer set: 5'-CCYTTCCTGGTGGCCCACACT (forward) and 5'-TTAGATACAAGCACAGTGYTG (reverse) (see primers A and B in Fig. 3). Reactions were subjected to 35 cycles of 94C for 30 sec, 55C for 30 sec, and 72C for 30 sec. No amplified products were observed in H_2_O or RT- controls (data not shown). The amplified 465 bp product was ligated into pCR3.1 (Invitrogen) following the manufacturer's instructions. Recombinant clones were screened by colony hybridization using the gel purified PCR product as probe. Plasmids were purified from hybridizing clones and sequenced using DyeTerminator Cycle sequencing (ABI). All hybridizing clones corresponded to inhibin α.

The full-length monkey inhibin α cDNA was amplified by RT-PCR from monkey testis RNA as described using a primer (5'-ATGGTGCTGCCCCTACTGCT) directed against the putative start of translation as determined by 5' rapid amplification of cDNA ends (RACE) (see below) and the reverse primer described above. Three prominent bands of approximately 1100, 700, and 400 bp were amplified. The top band was of the predicted size and was purified, cloned and sequenced. The identities of the other two bands have not yet been determined.

The mature region of the inhibin βB subunit cDNA was amplified from monkey (#1861) testis RNA by RT-PCR as described for the α subunit using the following primer set: 5'-AGCTGGCCGTGGTGCCBGTGTT (forward) and 5'-TCAGGCGCAGCCGCACTCCTC (reverse). The resulting 455 bp product was gel purified and sequenced directly.

### 5' RACE RT-PCR

5' RACE was performed on monkey (#1861) testis RNA using RNA ligase mediated (RLM)-RACE reagents following the manufacturer's instructions (Ambion, Austin, TX). The primary PCR was performed using the Outer Adapter Primer (Ambion) and the following gene specific primer: 5-GGCAGGTTTGGTGGGATGTGCA (Fig. 3, primer C). The secondary PCR was performed on 4 μl of a 1:100 dilution of the primary PCR reaction using the Inner Adapter Primer and the following nested gene specific primer: 5'-GGAAGGAGATGTTCAGTGCTAC (Fig. 3, primer D). For both PCR reactions, the following reaction conditions were used with AmpliTaq (Perkin-Elmer): 35 cycles of 94C for 30 sec, 55C for 30 sec, and 72C for 1.5 min. Three prominent amplicons were observed in the secondary PCR reaction. No bands were observed in the primary PCR or in any of the negative controls (data not shown). A pool of the different RACE products was ligated into pCR2.1 (Invitrogen). Plasmids were isolated from recombinant clones screened by α complementation and were sequenced as described.

### Northern blot

Twenty μg of total RNA prepared from testes of two adult rhesus monkeys were run on a 1% MOPS-formaldehyde agarose gel. RNA was transferred to Hybond N+ charged nylon membrane by capillary action using 20X SSC. The blot was first probed with a ^32 ^P-labeled (Ready-to-go; Amersham Pharmacia) cDNA corresponding to the last 465 bp of the coding sequence of monkey inhibin α. The blot was hybridized overnight at 42C in 50% formamide, 5X SSC, 1X Denhardt's, 20 mM NaPO_4 _(pH 6.8), 1% SDS, and 100 μg/ml denatured salmon sperm DNA using a modified sandwich method [19]. Following washes in 2X SSC/0.1% SDS at RT and 70C, the blot was exposed to X-ray film (Kodak) overnight with an intensifying screen at -85C. The blot was subsequently stripped and re-probed with a ^32 ^P-labeled cDNA corresponding to 256 bp of monkey inhibin α exon 1. Hybridization and washing conditions were as described for the first probe.

## Results

### Cloning of the rhesus monkey inhibin α cDNA

**Figure 1:**
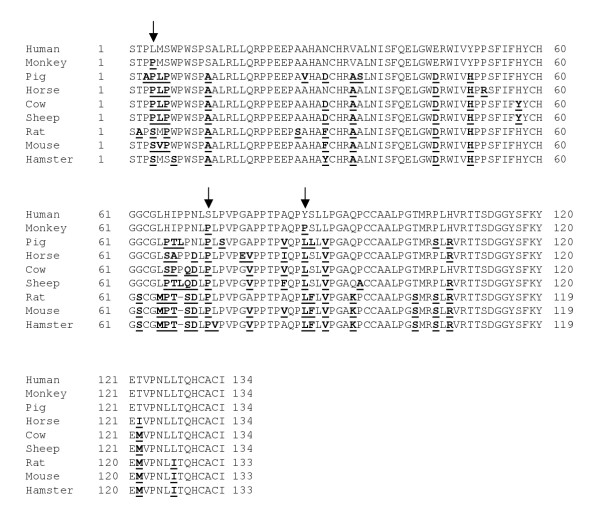
**Inhibin α amino acid sequence alignment. **Alignment of the inhibin α mature domain (αC) amino acid sequence in several mammalian species. Differences from the human sequence are bolded and underlined. Differences between the human and monkey sequences are indicated by arrows.

The inhibin α cDNA was cloned from rhesus monkey testis using a combination of RT-PCR based approaches. First, the cDNA encoding the last 20 amino acids of αN and the entirety of the mature (αC) domain was amplified by RT-PCR using adult monkey testis RNA as starting material. The resulting PCR product was cloned and sequenced. BLASTN of the non-redundant database showed highest sequence identity (97%) to the human inhibin α cDNA. Within the 402 bp encoding the αC domain, sequence identity was also 97% and the predicted amino acid sequence was 98% conserved (Fig. 1). Within the 134 amino acid αC domain there are three non-conservative differences between human and rhesus monkey (L4P, S72P, and Y86P; the first letter refers to the amino acid in human and the number denotes the residue in the αC domain); however, all occur at residues that vary between the mammalian inhibin α subunits sequenced thus far (Fig. 1). Proline at position 4 of rhesus monkey is also observed in pig, horse, cow and sheep. The proline at position 72 is observed in all mammalian species examined, except human. Finally, the proline at residue 86 is leucine in all non-human mammalian species examined thus far and is tyrosine in human.

To clone the full-length cDNA, we used 5' RACE to amplify the remainder of the αN and the pro-α regions. Three prominent RACE products were amplified. The longest (919–927 bp) and shortest (642 bp) were cloned and sequenced. The intermediate sized amplicon has not yet been definitively characterized. The long product was within the expected size range and was somewhat heterogeneous in that the clones had 5' ends that extended to differing extents. This likely reflects differences in transcription start sites, but all were within 8 bp of each other. The 5' untranslated region (UTR) ranged from 105 to 113 bp, which is slightly shorter than the 144 bp described in humans (GenBank acc.# NM_002191). When combined with the original PCR fragment, the contiguous sequence contained an open reading frame of 1098 bp predicted to encode a 366 amino acid prepro-hormone, consistent with the size of the human prepro-inhibin α.

**Figure 2:**
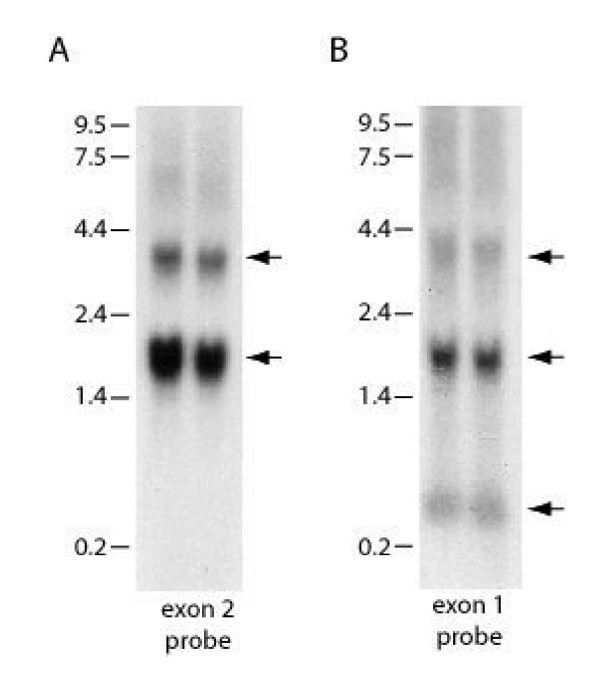
**Inhibin α mRNA expression in monkey testis. **Northern blots showing inhibin α mRNA expression in adult monkey testes. Equal amounts of total RNA from two adult males were run on a MOPS-formaldehyde gel. RNA was transferred to a nylon membrane and hybridized consecutively with ^32 ^P-labeled cDNA probes corresponding to 465 bp of exon 2 (A) and 256 bp of exon 1 (B) of monkey inhibin α. Both probes detected transcripts of 1.7 and 4 kb (top two arrows). The exon 1 probe also detected a 0.4 kb transcript (bottom arrow in B). Molecular weight standards (in kb) are shown at the left of each panel. Hybridization patterns were the same in both animals.

To confirm expression of a transcript containing this uninterrupted open-reading frame, PCR primers were designed against the start and end of the translation and the predicted 1101 bp fragment (including the stop codon) was amplified. DNA sequencing confirmed its identity. A monkey placental EST (CB548960) overlapped with and confirmed the sequence of the final 183 bp of the monkey inhibin α open reading frame and included an additional 188 bp of 3' UTR (not including the poly A+ tail). Thus, the mRNA encoding the inhibin α subunit would be predicted to be approximately 1.4 kb. Northern blot analysis of monkey testis RNA using a probe directed against sequence within αC domain (exon 2 probe) hybridized to an mRNA of approximately 1.7 kb (Fig. 2A). The slight size discrepancy may result from a long polyA+ tail, the use of alternative polyadenylation sequences (although a consensus AAUAAA sequence appears 21 nt upstream of the polyA+ tail in the EST), and/or alternative transcriptional start sites. An additional transcript of about 4 kb was also detected with this probe (top arrow in Fig. 2A). The identity of this less abundant transcript has not yet been ascertained. The monkey inhibin α sequence from the end of the longest 5'RACE product through the end of the open reading frame has been deposited in GenBank (GenBank Acc. #AY574369).

### Novel inhibin α mRNA in monkey testis

**Figure 4:**
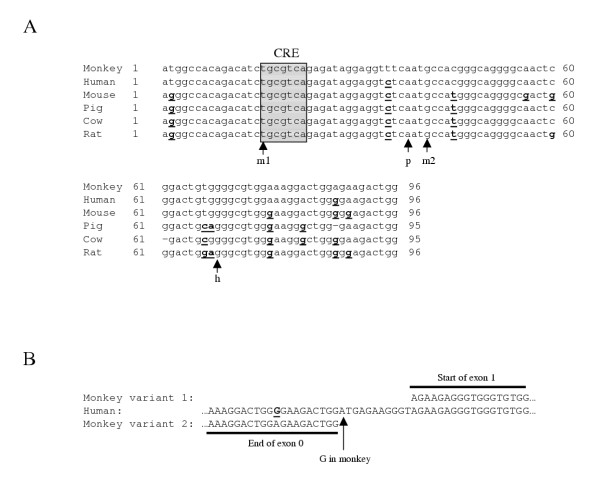
**DNA sequence alignment of novel exon 0 in monkey inhibin α. **A) Alignment of the novel exon 0 in inhibin α-variant 2 with inhibin α promoter sequences from Human (GenBank acc.# AF272341), mouse (GenBank acc.# M95526), pig (GenBank acc.# AF510728), cow (GenBank acc.# S72864), and rat (GenBank acc.# M32754). Note that the numbering is relative to the 96 bp of the monkey exon 0 and does not reflect the numbering in the GenBank entries. Bolded and underlined bases reflect differences from the monkey sequence and spaces (-) are added where needed to facilitate the alignment. The non-consensus cAMP responsive element (CRE), which is important for basal and FSH stimulated expression of inhibin α, is boxed and is conserved in all species. The arrows denote the start sites of the human (h, GenBank acc.# CB997542), mouse (m1, GenBank acc.# BY303064; m2, GenBank acc.# BI082792), and pig (p, GenBank acc.# BP457576) ESTs referred to in the text. B) Alignment of the end of monkey exon 0 and the start of exon 1 with human inhibin α genomic sequence. The AT dinucleotide in human is GT in monkey, thereby introducing a novel 5' splice donor site. The bold and underlined base reflects a difference from the monkey sequence.

The short RACE product when cloned and sequenced was determined to correspond to a novel variant of the inhibin α subunit. In humans (and other species), inhibin α has been described as a two exon gene (Fig. 3). Exon 1 encodes the 5' UTR, pro-α, and 85 bp of the αN region. The remainder of αN, αC and the 3'UTR are contained within exon 2. The 546 bp at the 3' end of the short RACE product corresponded exactly to sequence within monkey exon 2 (based on the human nomenclature). The 96 bp at the 5' end of the amplicon did not, however, correspond to the exon 1 sequence determined in the long RACE product. Upon BLASTN search, this 96 bp sequence was determined to show highest identity (91–97%) with human (GenBank acc.# AF272341), mouse (GenBank acc.# M95526), pig (GenBank acc.# AF510728), cow (GenBank acc.# S72864), and rat (GenBank acc.# M32754) inhibin α proximal promoter sequences (Fig. 4A). These data suggested that transcription of the short RACE product was initiated in what is conventionally thought of as inhibin α promoter (5' flanking sequence) in all species described to date.

We used this 96 bp sequence to screen the expressed sequence tag (EST) database to determine whether or not this portion of the inhibin α gene was included in transcripts in other species. BLASTN showed significant homology to four entries from three species: human placenta (GenBank acc.# CB997542), mouse dpc 14.5 Rathke's pouch (GenBank acc.# BY303064), mouse mammary tumor (GenBank acc.# BI082792), and pig ovary (GenBank acc.# BP457576). None of the ESTs extended as 5' as the monkey short RACE sequence, but one mouse EST (BY303064) started 15 bp 3' of where monkey RACE product began (Fig. 4A). In all cases, the EST sequences where contiguous with previously described 5' UTRs in exon 1 of the various species. Therefore, these ESTs appear to define alternative transcription initiation sites in the inhibin α gene and ostensibly increase the length of the 5' UTRs, but do not alter the exon-intron structure of the gene nor do they alter the open reading frame of the mRNA. This contrasts with what we observed in monkey.

We aligned both the short and long (see above) RACE product sequences to human genomic sequence derived from a BAC clone in GenBank (acc.# AC009955). The 96 bp unique to the short RACE product terminated 12 bp 5' of where the longest RACE product began (Fig. 4B). We noted that the first two bp of this intervening sequence was AT in human. We hypothesized that if the adenine in the first position in human was guanine in monkey, this would provide a 5' splice donor site (GT; [20]) and might explain how exon 1 sequence was skipped in its entirety in this transcript. We designed PCR primers corresponding to sequences flanking the intervening region and amplified genomic DNA extracted from monkey testis. Resulting amplicons of the predicted size were gel purified and sequenced directly. The results confirmed that the first two bp of the intervening sequence were GT in monkey, and therefore potentially provided a novel 5' splice site (Fig. 4B). The same 3' splice acceptor used in the long transcript also appears to be used in the short transcript, such that exon 2 is spliced identically in both cases (Fig. 3). We propose to call the unique sequence in the short RACE product exon 0. The resulting transcript reflects the splicing together of exon 0 and exon 2 (Fig. 3). Exon 1 and the intron are removed in the process. The long RACE product reflects transcription initiation from a downstream (more common?) site and is produced through splicing together of the canonical exon 1 and exon 2 (Fig. 3). We propose to call the transcript identified in the short RACE product rhesus monkey inhibin α-variant 2 (GenBank acc.# AY574370) and the canonical form inhibin α-variant 1.

The short RACE product was initiated from a reverse primer directed against sequence within exon 2 (Fig. 3, primer D). By virtue of its positioning, this primer excluded the last 287 bp of the open reading frame in exon 2. To confirm that inhibin α-variant 2 extended at least as far as the stop codon in exon 2, we used RT-PCR to amplify a contiguous sequence from the 5' end of exon 0 to the end of the ORF in exon 2. A faint band was amplified, but was of insufficient abundance to clone or directly sequence. We therefore performed nested PCR on this amplicon using primers directed against exon 0 (15 bp 3' of the first primer) and exon 2 (170 bp 5' of the first primer; primer C in Fig. 3). A band of the predicted size was amplified and directly sequenced following gel purification. The product corresponded to inhibin α-variant 2, indirectly confirming that the entirety of the open reading frame in exon 2 is contained within this transcript.

The ORF of inhibin-α variant 2 is 888 bp, potentially encoding a protein of 296 amino acids. A putative AUG start codon is observed 38 bp from the 5' end. However, the surrounding sequence does not conform to the consensus Kozak sequence [21]. The next AUG is observed 129 bp 3' of the first, within the αN encoding portion of exon 2. This potential start site also fails to conform to the consensus Kozak sequence. Therefore, it is not clear which, if either, of these codons may be used to initiate translation of inhibin α-short. The putative protein contains 19 novel amino acids (encoded by exon 0) at its N-terminus followed in-frame by amino acids 90–366 of inhibin α variant 1. The N-terminal 19 amino acid peptide does not encode a signal sequence nor does it possess significant homology to sequences in the public databases. The amino acids from exon 2 encode the majority of αN and the entirety of αC (see Fig. 3).

The northern blot in Fig. 2A was probed with a cDNA corresponding to exon 2, which is contained in both inhibin α-variants 1 and 2. Two transcripts were detected. To determine whether the transcripts might encode these two alternative forms, we stripped the blot and re-probed it with an exon 1 specific probe (Fig. 2B). Both transcripts were again detected, indicating that both contained exon 1 sequence and, by extension, that neither transcript encoded inhibin α-variant 2 (which lacks exon 1). This is perhaps not surprising in light of the difficulty we experienced in amplifying inhibin α-variant 2 by RT-PCR and is consistent with the notion that it is a relatively low abundance mRNA. Surprisingly, a smaller transcript of ~0.4 kb was also detected with the exon 1 probe. These data suggest that yet another inhibin α transcript may be expressed in monkey testis. The identity of this mRNA species is currently unknown; however, it is predicted to be truncated and contain some or all of exon 1 sequence.

### Cloning of the rhesus monkey inhibin βB cDNA

**Figure 5:**
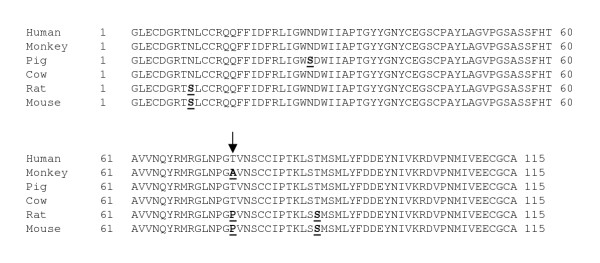
**Inhibin βB amino acid sequence alignment. **Alignment of the inhibin β_B _mature domain amino acid sequence in several mammalian species. Differences from the human sequence are bolded and underlined. An arrow indicates the one amino acid difference between the human and monkey sequences.

The mature domain of inhibin βB is highly conserved across all species investigated to date. We used RT-PCR to amplify this region of the cDNA in rhesus monkey (GenBank acc.# AY574371). Not surprisingly, the 115 amino acid domain shared 99% sequence identity with several other mammalian species, including human (Fig. 5). The one amino acid difference was a non-conservative threonine to alanine substitution at position 75 of the mature domain. This amino acid is also divergent (proline) in rat and mouse.

## Discussion

In this report, we describe the cloning of the inhibin B subunit cDNAs from testis of the adult rhesus monkey. The results indicate that both the inhibin α and inhibin βB subunits are highly conserved with other mammalian species, particularly within the mature domains of the prepro-hormones. For inhibin α, monkey and humans share 131 out of 134 amino acids. The three differences are all non-conservative; however, all occur at residues that vary across mammalian species. The human and monkey βB mature regions share 114 of 115 amino acids. Again, the one difference is non-conservative but occurs in a residue that varies across mammalian species. Collectively, these data suggest that the mature inhibin B is nearly identical in the human and rhesus macaque. As a result, current assays developed to measure human inhibin B are expected to accurately measure native monkey inhibin B. In addition, because rh-inhibin A and rh-inhibin B are equipotent at suppressing FSH secretion by monkey gonadotrophs in primary culture (Winters and Plant, unpublished observations), it seems reasonable to assume that the FSH suppressing potency of rh-inhibin A may be comparable to that of native monkey testicular inhibin B. If this assumption is substantiated when recombinant monkey inhibin B becomes available, then it will allow the physiological significance of previous and future studies using rh-inhibin A administration in male monkeys to be placed into perspective.

In the course of cloning the inhibin α subunit, we identified at least one alternative inhibin α mRNA, which we call rhesus monkey inhibin α-variant 2. The canonical inhibin α subunit mRNA, rhesus monkey inhibin α-variant 1, is comprised of two exons. Monkey inhibin α-variant 2 is produced through a combination of alternative transcription initiation and alternative splicing. As a result, a novel exon (exon 0) is used in place of exon 1 observed in inhibin α-variant 1. Both transcripts incorporate exon 2. 5' RACE indicated that transcription of inhibin α-variant 2 initiates about 108 bp 5' to the start of inhibin α-variant 1 (exon 1). As a result, the short variant contains an additional sequence at its 5' end that is conventionally referred to as inhibin α promoter or 5' flanking sequence. In monkey, an A to G transition (relative to the human sequence) in the genomic sequence upstream of the conventional transcription start site (in exon 1) leads to the introduction of a novel 5' splice donor site. As a result, exon 1 and the intron are removed from the pre-mRNA and a new exon (exon 0) is spliced to exon 2. The putative protein encoded by this variant is predicted to contain the majority of the αN and the entirety of the αC domains. At its N-terminus, however, it lacks a signal sequence as well as the pro-α region. Thus, if produced, it is unlikely that the protein would be secreted. We have not yet tried to express the protein to see if it is indeed synthesized in mammalian cells and where it may be trafficked. The putative translation initiation codon does not conform to the consensus sequence so there is some question about the efficiency with which this variant may be translated. In addition, its low abundance (at least at the mRNA level) also calls into question its functional significance.

At this point, we do not know to what extent inhibin α-variant 2 may be expressed in other species, nor have we examined its expression in monkey ovaries. However, this isoform may be unique to monkey because of the A to G transition (relative to human) in the genomic sequence. An alignment of the relevant "promoter" sequences in human, mouse, rat, and cow indicates that the splice donor (GT) in monkey is AT in human and GG in the other three species (not shown). Moreover, screening of the EST database with the monkey inhibin α-variant 2 unique sequence (exon 0) identified very few clones and in each case merely extended the 5' UTR in these species. That is, where monkey inhibin α-variant 2 skipped exon 1 entirely, the mouse, human, and pig EST sequences were contiguous with exon 1.

Perhaps the most important aspect of the identification of monkey inhibin α-variant 2 is that in this primate and perhaps in other species, transcription can be initiated further 5' than previously considered. As a result, sequences previously characterized as promoter may actually be 5' UTR, at least in some mRNA species. For example, a mouse EST contains sequence in its 5' UTR previously identified as a conserved non-consensus CRE in the inhibin α promoter [22] (Fig. 4A). Because this EST contains the CRE sequence, its transcription may not be cAMP dependent.

## Conclusions

Cloning of the inhibin B cDNAs from rhesus monkey testis indicates that the mature inhibin B is highly conserved in monkeys and humans. Therefore, the results in monkeys obtained with recombinant human inhibins may accurately reflect results that would be obtained with recombinant homologous ligands. The characterization of the inhibin subunit cDNAs in monkeys will greatly facilitate the production of macaque inhibins and will permit a direct test of this hypothesis. In addition, a novel inhibin α mRNA isoform was isolated in this investigation. This is the first example of an inhibin α mRNA variant described in any mammalian species. The results of both northern blot (Fig. 2) and RT-PCR analyses indicate that additional inhibin α mRNA variants also exist. Future studies will not only more thoroughly characterize these variants, but will examine their expression in other species. Moreover, functional analyses may highlight heretofore-unknown aspects of inhibin biology and function.

## Authors' contributions

DJB participated in the design of the study, performed all of the molecular biological experiments and analyses, and drafted significant portions of the manuscript. TKW participated in the design of the study and critically revised the manuscript. TMP provided the animal tissues, participated in the design of the study, and drafted sections of the manuscript. All authors read and approved the final manuscript.

## References

[B1] Gray PC, Bilezikjian LM, Vale W (2002). Antagonism of activin by inhibin and inhibin receptors: a functional role for betaglycan. Mol Cell Endocrinol.

[B2] Bernard DJ, Chapman SC, Woodruff TK (2001). Mechanisms of inhibin signal transduction. Recent Prog Horm Res.

[B3] Woodruff TK, D'Agostino J, Schwartz NB, Mayo KE (1988). Dynamic changes in inhibin messenger RNAs in rat ovarian follicles during the reproductive cycle. Science.

[B4] Bernard DJ, Woodruff TK (2001). Inhibin binding protein in rats: alternative transcripts and regulation in the pituitary across the estrous cycle. Mol Endocrinol.

[B5] Woodruff TK, Besecke LM, Groome N, Draper LB, Schwartz NB, Weiss J (1996). Inhibin A and inhibin B are inversely correlated to follicle-stimulating hormone, yet are discordant during the follicular phase of the rat estrous cycle, and inhibin A is expressed in a sexually dimorphic manner. Endocrinology.

[B6] Illingworth PJ, Groome NP, Duncan WC, Grant V, Tovanabutra S, Baird DT, McNeilly AS (1996). Measurement of circulating inhibin forms during the establishment of pregnancy. J Clin Endocrinol Metab.

[B7] Chapman SC, Woodruff TK (2003). Betaglycan localization in the female rat pituitary: implications for the regulation of follicle-stimulating hormone by inhibin. Endocrinology.

[B8] Plant TM, Padmanabhan V, Ramaswamy S, McConnell DS, Winters SJ, Groome N, Midgley A. R., Jr., McNeilly AS (1997). Circulating concentrations of dimeric inhibin A and B in the male rhesus monkey (Macaca mulatta). J Clin Endocrinol Metab.

[B9] Groome NP, Illingworth PJ, O'Brien M, Pai R, Rodger FE, Mather JP, McNeilly AS (1996). Measurement of dimeric inhibin B throughout the human menstrual cycle. J Clin Endocrinol Metab.

[B10] Hayes FJ, Pitteloud N, DeCruz S, Crowley W. F., Jr., Boepple PA (2001). Importance of inhibin B in the regulation of FSH secretion in the human male. J Clin Endocrinol Metab.

[B11] Schwall RH, Mason AJ, Wilcox JN, Bassett SG, Zeleznik AJ (1990). Localization of inhibin/activin subunit mRNAs within the primate ovary. Mol Endocrinol.

[B12] Roberts VJ, Barth S, el-Roeiy A, Yen SS (1993). Expression of inhibin/activin subunits and follistatin messenger ribonucleic acids and proteins in ovarian follicles and the corpus luteum during the human menstrual cycle. J Clin Endocrinol Metab.

[B13] Anawalt BD, Bebb RA, Matsumoto AM, Groome NP, Illingworth PJ, McNeilly AS, Bremner WJ (1996). Serum inhibin B levels reflect Sertoli cell function in normal men and men with testicular dysfunction. J Clin Endocrinol Metab.

[B14] Robertson DM, Cahir N, Findlay JK, Burger HG, Groome N (1997). The biological and immunological characterization of inhibin A and B forms in human follicular fluid and plasma. J Clin Endocrinol Metab.

[B15] Winters SJ, Plant TM (1999). Partial characterization of circulating inhibin-B and pro-alphaC during development in the male rhesus monkey. Endocrinology.

[B16] McNeilly AS, Souza CJ, Baird DT, Swanston IA, McVerry J, Crawford J, Cranfield M, Lincoln GA (2002). Production of inhibin A not B in rams: changes in plasma inhibin A during testis growth, and expression of inhibin/activin subunit mRNA and protein in adult testis. Reproduction.

[B17] Majumdar SS, Mikuma N, Ishwad PC, Winters SJ, Attardi BJ, Perera AD, Plant TM (1995). Replacement with recombinant human inhibin immediately after orchidectomy in the hypophysiotropically clamped male rhesus monkey (Macaca mulatta) maintains follicle-stimulating hormone (FSH) secretion and FSH beta messenger ribonucleic acid levels at precastration values. Endocrinology.

[B18] Ramaswamy S, Pohl CR, McNeilly AS, Winters SJ, Plant TM (1998). The time course of follicle-stimulating hormone suppression by recombinant human inhibin A in the adult male rhesus monkey (Macaca mulatta). Endocrinology.

[B19] Wu S, Lu Q, Kriz AL (1995). Multiple-sandwich, one-step hybridization of northern and Southern blots. Biotechniques.

[B20] Konarska MM (1998). Recognition of the 5' splice site by the spliceosome. Acta Biochim Pol.

[B21] Kozak M (1987). An analysis of 5'-noncoding sequences from 699 vertebrate messenger RNAs. Nucleic Acids Res.

[B22] Pei L, Dodson R, Schoderbek WE, Maurer RA, Mayo KE (1991). Regulation of the alpha inhibin gene by cyclic adenosine 3',5'-monophosphate after transfection into rat granulosa cells. Mol Endocrinol.

